# Predictors of peer victimisation in the context of secondary school

**DOI:** 10.1080/07853890.2021.1896153

**Published:** 2021-09-28

**Authors:** Patrícia Gouveia, Isabel Leal, Lara Neves, Jorge Cardoso

**Affiliations:** a Laboratório de Psicologia Egas Moniz (LabPSI), Centro de Investigação Interdisciplinar Egas Moniz (CiiEM), Egas Moniz Cooperativa de Ensino Superior, Caparica, Portugal; b ISPA, Instituto Universitário, William James Center for Research (WJCR), Lisbon, Portugal; c Liga de Amigos do Hospital Garcia de Orta (LAHGO), Almada, Portugal

## Abstract

**Introduction:**

Peer victimisation is defined as the experience of being the target of aggressive behaviour, by one or more peers. This presents several configurations, and in this sense the study of multiple types of victimisation is emphasised [1]. Research supports the need of studying protective and risk factors for victimisation in light of the ecological model, once it highlights the connection between individual characteristics, family, school and community [2]. Two main objectives will be analysed: (a) the risk factors associated with four global types of victimisation (overt, reactive overt, relational and reactive relational) and seven subtypes of victimisation behaviours that the adolescent’s might experience (e.g. social manipulation, social exclusion) using the ecological model; (b) discuss how the study of risk factors contributes to the strategies to be outlined in the context of an intervention program.

**Materials and methods:**

584 high school adolescents participated in this quasi-experimental study, aged between 12 and 19 years (*M* = 15.20; DP = 1.90). The participants completed: School aggression and violence: Victimisation symptoms and behaviours Questionnaire [3], Perception of Social-Family Support and Perception of Peer Support Scales [4], Parental Support Questionnaire – Short Form [5], and School Climate Questionnaire [6]. Ethics committee approved the investigation and informed consent was obtained from parents. The questionnaires (anonymous and confidential) were completed in a classroom context. Using Hierarchical Linear Regression, two models were built, in which the qualitative variables (e.g. age, gender) were first inserted, followed by the factors peer support, family and school climate.

**Results:**

The global types of victimisation were better explained by reactive relational victimisation (18.60% of total variance) while the specific types of victimisation by the harassment model (19.30% of the total variance), both by the factor social support from family and friends (respectively 13.80% and 10.70% of the variance). In short, boys showed a higher risk of overt victimisation, while girls reported more relational victimisation. Perceiving a poor support from family and friends places the adolescents at a higher risk of peer victimisation by harassment, but also by exclusion/social isolation, coercion, intimidation, threats and aggressiveness. The higher the perception of a school climate characterised by violence, the higher the risk of peer victimisation, specifically by harassment. No cases of victimisation per bullying were identified.

**Discussion and conclusions:**

The risk factors found were indicative that these adolescents would benefit from a universal intervention model. These are developed in the classroom context for all students and do not include a selection of subjects with a specific need for intervention. Strategies should be oriented towards socio-emotional interventions, which include emotional management and interpersonal conflicts. These have shown good results in reducing violence in young people, including bullying, but also in reducing disruptive behaviours in the classroom. Greater knowledge of teachers and administrative staff, but also parents, and using rules that promote social inclusion, would facilitate the identification and signalling of students at risk. School administrations should adopt more integrated prevention policies and not only sanctioning measures, promoting a school climate perceived as protective and without violence.

## References

[1] Berkowitz R, De Pedro KT, Gilreath TD. A latent class analysis of victimization among middle and high school students in California. J School Violence. 2014; 14:1–18.

[2] Ferrer BM, Ruiz DM, Amador LV, et al. School victimization among adolescents. An analysis from an ecological perspective. Psychological Intervention. 2011;20(2):149–160.

[3] Piñuel I, Oñate A. Agressão e Violência Escolar – Comportamentos e Sintomas de Vitimização (AVE). Portuguese adaptation: Patrícia Gouveia and Lara Neves. Lisbon: Hogrefe; 2017.

[4] Gouveia P, Leal I, Cardoso J, et al. Contributos para a adaptação e aferição de duas medidas de suporte Social. Psicol Saúde Doenças. 2015;16(3):386–391.

[5] Gouveia P, Leal I, Cardoso J. Adaptação do Questionário de Suporte Parental (QSP-6) – Versão reduzida para adolescentes. Psicol Saúde Doenças. 2015;16:189–198.

[6] Scherman V. School climate instrument: a pilot study in Pretoria and environs [Dissertation, Master’s in Research Psychology]. Faculty of Humanities of the University of Pretoria; 2005.

